# Insulin Secretion and Risk for Future Diabetes in Subjects with a Nonpositive Insulinogenic Index

**DOI:** 10.1155/2018/5107589

**Published:** 2018-03-22

**Authors:** Daisuke Aono, Rie Oka, Mitsuhiro Kometani, Yoshimichi Takeda, Shigehiro Karashima, Kenichi Yoshimura, Yoshiyu Takeda, Takashi Yoneda

**Affiliations:** ^1^Department of Cardiovascular and Internal Medicine, Kanazawa University Graduate School of Medicine, Kanazawa, Japan; ^2^Department of Internal Medicine, Hokuriku Central Hospital, Oyabe, Japan; ^3^Department of Biostatistics, Innovative Clinical Research Center (iCREK), Kanazawa University Hospital, Kanazawa, Japan

## Abstract

**Aim:**

To characterize subjects with a nonpositive insulinogenic index and longitudinally observe changes in their glucose tolerance.

**Subjects and Methods:**

A historical cohort study was conducted using data from the medical checkups of public school workers. Indices of insulin secretion and insulin sensitivity derived from oral glucose tolerance test (OGTT) and the incidences of diabetes and impaired glucose tolerance (IGT) were compared among subgroups of subjects with different insulinogenic index (change in insulin/change in glucose over the first 30 min on the OGTT).

**Results:**

Of the 1464 nondiabetic subjects at baseline, 72 (4.9%) subjects had a nonpositive insulinogenic index: 42 of those subjects had a nonpositive glucose response (ΔGlu_0–30_ ≤ 0) and 30 had a nonpositive insulin response (ΔIns_0–30_ ≤ 0). Compared with subjects who had normal glucose tolerance (NGT) with insulinogenic index ≥ 0.4, subjects with a nonpositive glucose response had a higher first-phase Stumvoll and lower incidences of diabetes and IGT based on a log-rank test (*p* < 0.05), whereas subjects with a nonpositive insulin response had lower indices of insulin secretion and a higher incidence of diabetes (*p* < 0.05).

**Conclusions:**

These results demonstrate that in the first 30 min on the OGTT, subjects with a nonpositive insulinogenic index due to a nonpositive glucose response (ΔGlu_0–30_ ≤ 0) had a lower risk for future diabetes and that subjects with nonpositive insulin response (ΔIns_0–30_ ≤ 0) had a higher risk for future one.

## 1. Introduction

Insulinogenic index (change in insulin/change in glucose over the first 30 min after the load) on the oral glucose tolerance test (OGTT) has been widely used as an index of early phase insulin secretion in clinical studies [1–4]. It is highly correlated with the acute insulin response (AIR) on intravenous glucose tolerance test (IVGTT) and is considered a reasonable surrogate [5, 6]. Measuring insulinogenic index has several advantages; it involves less complex protocols, needs less costs, and utilizes a physiological route of glucose administration [7, 8]. Simpler methods for the assessment of insulin secretion are those based on basal glucose and insulin concentrations, such as HOMA-beta [9], but fasting levels cannot provide insight into the secretory response of beta cells to rising glucose concentrations [8]. Indeed, insulinogenic index or its composite with insulin sensitivity (oral disposition index) has been demonstrated to be predictive of type 2 diabetes, independent of fasting plasma glucose in different populations [4, 10–12].

Sometimes, however, a small number of subjects in general populations exhibit a nonpositive insulinogenic index due to either a decrement in insulin or in glucose value 30 min after glucose ingestion from fasted states [13]. Most studies, including ours, exclude them from the main analysis [14]. Abdul-Ghani et al. have reported a lower risk for future diabetes in individuals whose plasma glucose concentrations returned to equal to or below fasting levels within 30 min compared with those who required longer time to return their plasma glucose to fasting levels [15]. Besides the report by Abdul-Ghani et al., there has been no report on the clinical characteristics and the risk for future diabetes in subjects with a nonpositive insulinogenic index.

The aim of this study, therefore, was to characterize subjects with a nonpositive insulinogenic index and longitudinally observe changes in their glucose tolerance.

## 2. Methods

### 2.1. Study Participants

A historical cohort study was conducted using data from the medical checkups of public school employees collected at Hokuriku Central Hospital [14]. During April 2006 and March 2010 (baseline period), 2340 employees underwent an OGTT at a medical checkup [16]. If employees received more than one checkup during the baseline period, the initial checkup data were used. After those who had fasting plasma glucose ≥ 126 mg/dL and/or 2 h plasma glucose ≥ 200 mg/dL (*n* = 85), those who had HbA1c values ≥ 6.9% (52 mmol/mol) (*n* = 42), those who had undergone gastrectomy (*n* = 32), those who were taking steroids (*n* = 1) or anticancer drugs (*n* = 1), those who or had any missing data (*n* = 18) were excluded, 2161 nondiabetic individuals were selected. Of these, 1464 individuals repeated checkups at least once until March 2016 and comprised our study sample. The remaining 697 participants did not have a repeat checkup, yielding a follow-up rate of 67.7%. If the subjects developed diabetes, the follow-up ended at that time. An OGTT was performed at all checkups during the follow-up period; however, within the last two years (April 2014 to March 2016), an OGTT was performed on 1191 participants and the measurement of fasting plasma glucose only was performed in the remaining 273 participants for financial reasons. Information on smoking and drinking habits and a medical history were obtained via a questionnaire. Participants were considered current smokers if they smoked at least one cigarette per day. Alcohol use was defined by the number of days per week it was consumed, regardless of the amount. Informed consent was obtained by an opt-out method, and the institutional review board of the Kanazawa University approved the study protocol on June 21, 2017 (IRB number 2497-1); the study protocol conforms to the provisions of the Declaration of Helsinki. The study was registered on the University Hospital Medical Information Network Clinical Trials Registry (UMIN-CTR, http://www.umin.ac.jp/ctr, UMIN ID: UMIN000017662).

### 2.2. Blood Sampling and Assay

All evaluations were performed at the Health Check Department of the Hokuriku Central Hospital. Participants were asked to visit the hospital between 8:00 a.m. and 9:00 a.m. after an overnight fast. At the baseline visit, an OGTT (75 g dextrose monohydrate in 250 mL water) with 0, 30, 60, and 120 min sampling to determine plasma glucose and insulin concentrations was performed on all participants [16]. Plasma glucose was assessed using the glucose oxidase method (Automatic Glucose Analyzer ADAMS Glucose GA-1160, Arkray, Kyoto, Japan) at the hospital laboratory. Insulin concentration assays were performed by the chemiluminescence immunoassay method at a commercial laboratory (BML Inc., Tokyo, Japan), with an intra-assay coefficient of variation (CV) of 2.4–3.2% based on 10 replicates of 3 different samples. Height and weight were measured, and body mass index (BMI) was calculated by dividing weight (kg) by height squared (m^2^).

### 2.3. Calculations of Indices of Insulin Secretion and Insulin Sensitivity

The indices of insulin secretion used in this study were as follows: insulinogenic index = (Ins_30_ − Ins_0_)/(Gluc_30_ − Gluc_0_) or ΔIns_0–30_/ΔGlu_0–30_, where Ins*_y_* and Glu*_y_* represent values at time (*y*: min) during the OGTT [5, 6]; the ratio of area under the curve (AUC) for insulin to AUC for glucose for the specific time frame, which was calculated by applying the trapezoid rule; first-phase Stumvoll = 1283 + 1.829 × Ins_30_–138.7 × Glu_30_+ 3.772 × Ins_0_; and second-phase Stumvoll = 286 + 0.416 × Ins_30_–25.94 × Glu_30_+ 0.926 × Ins_0_ [17]. The indices of insulin sensitivity were as follows: Matsuda insulin sensitivity index (ISI) = 10,000/(Glu_0_ × Ins_0_ × Glu_120_ × Ins_120_)^0.5^ [18] and homeostatic model assessment of insulin resistance (HOMA-IR) = Glu_0_ × Ins_0_/405 [9]. We used different units for glucose concentration and insulin concentration in calculating each index, as described in Table 1.

### 2.4. Statistical Analysis

The data are presented as the mean ± standard deviation or median with interquartile ranges for continuous variables or as a frequency for categorical variables. Continuous variables were compared using one-way analysis of variance (ANOVA) or Kruskal-Wallis test, and categorical values were compared using a *χ*^2^ test, with a Bonferroni correction for multiple comparisons. The participants were classified by their insulinogenic index at 0.4 because this threshold was adopted by the Japan Diabetes Society to identify individuals at a high risk of progression to diabetes [19]. Participants were further classified by their glucose tolerance using the criteria of the American Diabetes Association (ADA) [20]; subjects were categorized as having NGT (fasting plasma glucose < 100 mg/dL and 2 h plasma glucose < 140 mg/dL), impaired fasting glucose (IFG) (fasting plasma glucose = 100–125 mg/dL), and impaired glucose tolerance (IGT) (2-hour plasma glucose = 140–199 mg/dL). Among groups, indices of insulin sensitivity and insulin secretion were compared using analysis of covariance (ANCOVA) with age, gender, and BMI as covariates. Insulinogenic index and HOMA-IR were log-transformed using the natural log prior to analysis to reduce skewness; the transformed values produced a good fit to normal distribution. Finally, the progression to diabetes defined as fasting plasma glucose ≥ 126 mg/dL, 2-hour plasma glucose ≥ 200 mg/dL, and/or taking treatments for diabetes or to IGT among subjects grouped by insulinogenic index was compared by the Kaplan-Meier method. The analysis of the progression to IGT was performed in 982 out of the 1169 NGT subjects at baseline, because the remaining 187 NGT subjects did not receive an OGTT at follow-up. A *p* value of < 0.05 was considered statistically significant. All analyses were conducted using SPSS software version 24.0 for Windows (SPSS Inc., Chicago, USA).

## 3. Results

**Figure 1:**
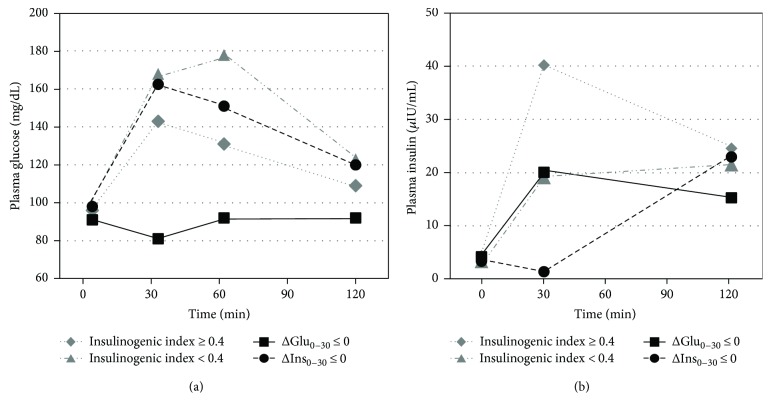
Plasma glucose and insulin concentrations on the OGTT among subgroups of subjects with a different insulinogenic index as presented as median values.

Study subjects were 1464 nondiabetic individuals with a mean age of 52.0 years and a mean BMI of 23.9 kg/m^2^ at baseline. There were 42 (2.9%) subjects whose plasma glucose concentrations fell equal to or below their fasting levels at 30 min (ΔGlu_0–30_ ≤ 0) and 30 (2.0%) subjects whose insulin concentrations fell equal to or below the fasting levels at 30 min after the oral glucose load (ΔIns_0–30_ ≤ 0). Three subjects with double nonpositive values, resulting in a positive insulinogenic index, were excluded from the following analysis. As shown in Figure 1, subjects with ΔIns_0–30_ ≤ 0 increased their insulin concentrations at 120 min from 30 min, while those with ΔGlu_0–30_ ≤ 0 got to return their insulin concentrations at 120 min. As shown in Table 2, subjects with ΔGlu_0–30_ ≤ 0 included a lower proportion of men and had a lower BMI and lower glucose and insulin concentrations during OGTT, compared with subjects with insulinogenic index ≥ 0.4 (*p* < 0.05). Subjects with ΔIns_0–30_ ≤ 0 had a higher BMI and higher glucose and fasting insulin concentrations compared with subjects with ΔGlu_0–30_ ≤ 0 (*p* < 0.05). Subjects with ΔGlu_0–30_ ≤ 0 had a significantly higher proportion of NGT (92.9%) compared with the other three groups listed in Table 2 (*p* < 0.05).

To evaluate insulin secretion and insulin sensitivity on the basis of the OGTT, we classified subjects with nonpositive insulinogenic index into 2 groups (i.e., ΔGlu_0–30_ ≤ 0 and ΔIns_0–30_ ≤ 0) and those with positive insulinogenic index into 2 groups as well [with subgroups] (i.e., NGT [insulinogenic index ≥ 0.4 or <0.4] and IFG and/or IGT [insulinogenic index ≥ 0.4 or <0.4]) (Table 1). In subjects with ΔGlu_0–30_ ≤ 0, indices of insulin secretion such as AUC(Ins_30_)/AUC(Glu_30_) and AUC(Ins_120_)/AUC(Glu_120_) were not significantly decreased and first-phase Stumvoll was increased compared with NGT with insulinogenic index ≥ 0.4. In subjects with ΔIns_0–30_ ≤ 0, all indices of insulin secretion examined were significantly decreased compared with IFG and/or IGT as well as NGT with insulinogenic index ≥ 0.4. Matsuda ISI and HOMA-IR were not significantly different in subgroups with nonpositive insulinogenic index compared with NGT with insulinogenic index ≥ 0.4. These indices were estimated after adjustments for gender, age, and BMI; however, the results were essentially the same before the adjustments (data not shown).

**Figure 2:**
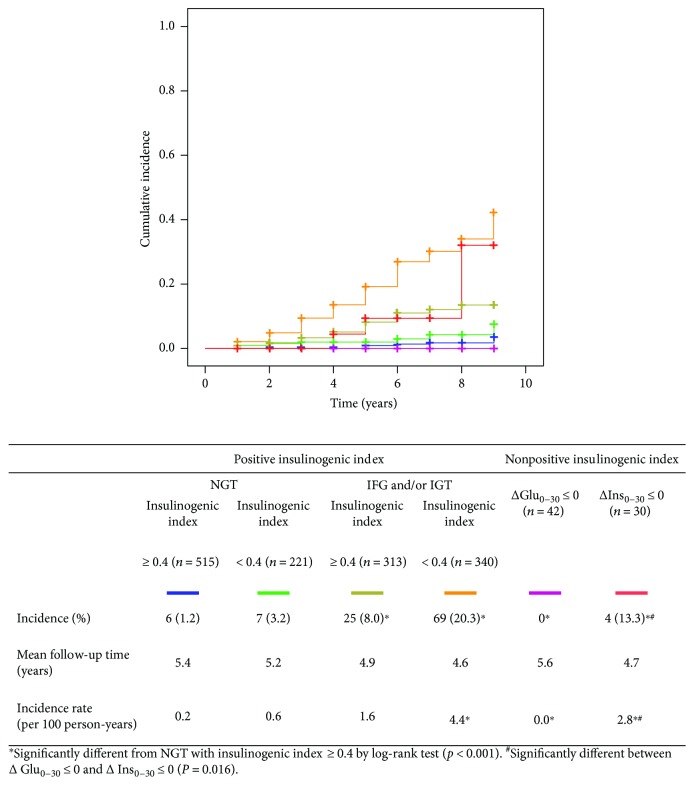
Cumulative incidence of diabetes by the Kaplan-Meier method among groups by insulinogenic index.

**Figure 3:**
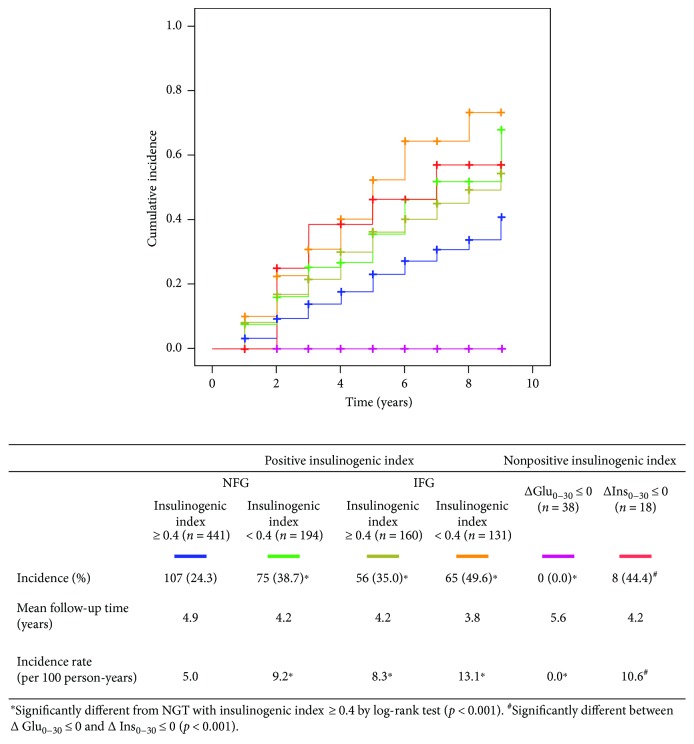
Cumulative incidence of impaired glucose tolerance or type 2 diabetes (2-hour PG ≥ 140 mg/dL) by the Kaplan-Meier method among groups by insulinogenic index.

The progression to diabetes (Figure 2) or to IGT (Figure 3) was assessed among different subgroups by insulinogenic index. In subjects with ΔGlu_0–30_ ≤ 0, there was no progression to diabetes nor to IGT during a mean follow-up period of 5.6 years, which was significantly less than NGT with insulinogenic index ≥ 0.4 by log-rank test (*p* < 0.05). In subjects with ΔIns_0–30_ ≤ 0, the incidence of diabetes was significantly higher compared with NGT with insulinogenic index ≥ 0.4 (*P* < 0.05) while that of IGT was not statistically different by log-rank test. In comparison between subjects with ΔGlu_0–30_ ≤ 0 and those with ΔIns_0–30_ ≤ 0, the incidences of diabetes and of IGT were significantly different (*p* = 0.016 and *p* < 0.001, resp.).

## 4. Discussion

In this study, subjects with a nonpositive insulinogenic index were characterized based on insulin secretion and insulin sensitivity and diabetes and IGT incidences were compared with subjects who had a positive insulinogenic index. Although the prevalence of nonpositive insulinogenic index was low in agreement with prior studies investigating Western cohorts [13], this is the first study in Asian populations that longitudinally observed the future incidence of glucose intolerance in subjects with a nonpositive insulinogenic index. The findings of the current study could be useful for estimating the risk for diabetes when clinicians encounter subjects with a nonpositive insulinogenic index.

The prevalence of a nonpositive insulinogenic index was 4.9% (72/1464) in nondiabetic subjects in this study. The prevalence in other cohorts has been reported as 8.2% in the San Antonio Heart Study, 3.4% in the Genetics of NIDDM Study, and 2.5% in the Japanese American Community Diabetes Study [13]. There were two reasons for a nonpositive insulinogenic index: a nonpositive glucose response (ΔGlu_0–30_ ≤ 0) and a nonpositive insulin response (ΔIns_0–30_ ≤ 0) after a glucose load. The former included a higher proportion of NGT than the latter in this study (92.9% versus 46.7%), which is in agreement with the results from the San Antonio Heart Study (81.4% versus 11.8%). We confirmed and extended the observation that a nonpositive insulinogenic index occurs at a given frequency in Japanese populations.

Subjects with a nonpositive glucose response 30 min after a glucose load had similar or even higher insulin secretion compared with NGT with insulinogenic index ≥ 0.4 and a lower risk for future deterioration of glucose tolerance. Abdul-Ghani et al. have reported that individuals whose plasma glucose concentrations return to equal to or below the fasting levels by 30 min after a glucose load showed the highest insulin sensitivity, the highest insulin secretion, and the lowest risk for future diabetes, compared with those who required >30 min for their plasma glucose to return to fasting levels in Mexican Americans [15] and Caucasians [21]. Of note, the insulinogenic index could not be calculated due to nonpositive glucose response in their study, so they assessed insulin secretion by AUC (Ins_30_)/AUC(Glu_30_), which was also applied in this study. The subjects with a nonpositive glucose response are likely to be highly efficient in disposing glucose and in maintaining normal glucose homeostasis following a glucose load.

Subjects with a nonpositive insulin response 30 min after the glucose load had the lowest insulin secretion among different groups of the insulinogenic index. This group comprised of more IFG and/or IGT and showed a higher BMI compared with the abovementioned subjects with a nonpositive glucose response. In the study examining the acute (first-phase) insulin response to intravenous glucose, a paradoxically nonpositive insulin response had also been observed in subjects with fasting hyperglycemia [22]. A nonpositive insulin response at 30 min on the OGTT may reflect an impairment of early insulin response, which is one of the primary pathophysiologic changes of type 2 diabetes.

Of note, insulin levels at 120 min in subjects with a nonpositive insulin response were similar to other groups, indicating that insulin secretion was delayed but preserved (Figure 1). Recently, Lorenzo et al. have reported that indices of insulin secretion derived from the late OGTT period as well as the early period (e.g., insulinogenic index) independently predict the incidence of diabetes from the San Antonio Study [23]. Although the late insulin secretion may keep the subjects with a nonpositive insulin response from the development of diabetes, they are likely to have a higher risk of diabetes compared with the IFG and/or IGT with insulinogenic index ≥ 0.4.

We evaluated several OGTT-based indices of insulin secretion. When glucose is administered intravenously, the insulin response to a hyperglycemic stimulation is biphasic [24]; plasma insulin concentrations increase rapidly to a peak at 2 to 4 min, decrease within 10 to 15 min (first-phase insulin release), and gradually increase over 120 min (second-phase insulin release) [25]. First-phase Stumvoll has been validated against this first-phase insulin release on hyperglycemic clamp test [26]. Although measurements of plasma insulin are ordinarily performed at 30 min on the OGTT, this time point may be too late to capture the early insulin response in some subjects with a prompt secretory response to glucose stimulation. In these subjects, the first burst of insulin secreted into the portal vein would suppress hepatic glucose production and decrease their plasma glucose equal to or below the fasting levels by 30 min after the glucose load.

The strength of our study is the longitudinal design, which allows for a comparison of future risk for diabetes among groups by insulinogenic index. However, several limitations of this study should be considered. First, the follow-up rate of the subjects was 67.7%. Subjects who missed checkups during the follow-up period might be less conscious about their health, which biased the study subjects toward metabolically healthy people. However, baseline characteristics including BMI, glucose, and insulin concentrations between the subjects who were and were not followed were not significantly different. Second, measurements of C-peptide were not performed. C-peptide has been utilized to assess the insulin secretory function of beta cells because it is not degraded by the liver and thus represents prehepatic insulin secretion [27]. However, in estimation of the prehepatic insulin secretion, the calculation from insulin and that from C-peptide has been shown to be highly correlated (*R*^2^ = 0.75, *p* < 0.001) [28]. Third, the measurement of plasma glucose concentrations relied on a single OGTT, which is known to have within-subject variability [29].

In conclusion, a nonpositive insulinogenic index occurs at a given frequency in Japanese populations, due to a nonpositive glucose response or due to a nonpositive insulin response 30 min after the load. The former is efficient in disposing glucose and has a lower risk for future diabetes, while the latter showed a lower early insulin response and has a higher risk for future diabetes.

## Figures and Tables

**Table 1:** Indices of insulin secretion and insulin sensitivity among groups by insulinogenic index.

	Positive insulinogenic index				Nonpositive insulinogenic index	
	NGT		IFG and/or IGT		ΔGlu_0–30_ ≤ 0 (*n* = 42)	ΔIns_0–30_ ≤ 0 (*n* = 30)
	Insulinogenic index ≥ 0.4 (*n* = 515)	Insulinogenic index < 0.4 (*n* = 221)	Insulinogenic index ≥ 0.4 (*n* = 42)	Insulinogenic index < 0.4 (*n* = 340)		
Insulin secretion						
Insulinogenic index (mU/L/mg/dL)	0.92 (0.87–0.97)†	0.24 (0.22–0.26)^∗^†	0.79 (0.73–0.84)^∗^	0.20 (0.18–0.21)^∗^†	Not calculable	Not calculable
AUC (Ins_30_)/AUC (Glu_30_) (pmol/mmol)	27.6 (26.6–28.7)	12.7 (11.2–14.2)^∗^†	26.7 (25.4–28.0)	10.9 (9.6–12.1)^∗^†	24.3 (20.8–27.8)	3.8 (−0.4–7.9)^∗^†
AUC (Ins_120_) (nmol^∗^min)	28.4 (27.3–29.7)†	17.0 (15.2–18.8)^∗^†	34.5 (33.0–36.1)^∗^	19.4 (17.9–20.9)^∗^†	20.0 (15.9–24.1)^∗^†	10.4 (5.6–15.3)^∗^†
AUC (Ins_120_)/AUC (Glu_120_) (×10^−2^ mU/mg)	2.79 (2.69–2.90)	1.41 (1.25–1.57)^∗^†	2.86 (2.72–2.99)	1.37 (1.25–1.50)^∗^†	2.51 (2.16–2.87)	0.85 (0.43–1.27)^∗^†
First-phase Stumvoll (pmol/L, mmol/L)	945 (918–972)†	464 (423–506)^∗^†	853 (818–888)^∗^	276 (243–309)^∗^†	1156 (1062–1251)^∗^†	232 (121–343)^∗^†
Second-phase Stumvoll (pmol/L, mmol/L)	254 (248–260)	150 (141–159)^∗^†	240 (232–248)	115 (107–122)^∗^†	285 (264–306)†	99 (74–124)^∗^†
Insulin sensitivity						
Matsuda insulin sensitivity index (mU/L, mg/dL)	14.0 (13.0–15.1)†	18.4 (16.8–20.0)^∗^†	10.1 (8.7–11.4)^∗^	10.7 (9.4–12.0)^∗^	17.2 (13.6–20.9)†	14.5 (10.1–18.8)
HOMA-IR (mU/L, mmol/L)	0.89 (0.85–0.93)†	0.70 (0.65–0.76)^∗^†	1.13 (1.06–1.20)^∗^	0.92 (0.87–0.98)†	0.87 (0.73–1.03)	0.88 (0.73–1.08)

Data are means (95% confidence intervals) adjusted for body mass index, age, and gender. Insulinogenic index and HOMA-IR were log-transformed before analysis, and calculated values were untransformed after analysis. ^∗^*p* < 0.05 compared to NGT with insulinogenic index ≥ 0.4 and †*p* < 0.05 compared to IFG and/or IGT with insulinogenic index ≥ 0.4 with Bonferroni correction for multiple comparisons. AUC(Glu_time_): area under the curve for glucose by time (min) elapsed; AUC(Ins_time_): area under the curve for insulin by time (min) elapsed; ΔGlu_0–30_ and ΔIns_0–30_,: change in glucose and in insulin, respectively, over the first 30 min on the oral glucose tolerance test; HOMA-IR: homeostasis model of insulin resistance.

**Table 2:** Baseline characteristics by level of the insulinogenic index.

	Positive insulinogenic index		Nonpositive insulinogenic index	
	Insulinogenic index ≥ 0.4 (*n* = 828)	Insulinogenic index < 0.4 (*n* = 561)	ΔGlu_0–30_ ≤ 0 (*n* = 42)	ΔIns_0–30_ ≤ 0 (*n* = 30)
Age (years)	51.6 ± 7.0	52.9 ± 6.7^∗^	50.6 ± 7.1	50.2 ± 8.5
Male gender (%)	65.3	79.5^∗^	42.9^∗^†	63.3
Body mass index (kg/m^2^)	24.3 ± 3.3	23.6 ± 2.9^∗^	22.4 ± 2.7^∗^	24.8 ± 4.3‡
Fasting plasma glucose (mg/dL)	92/96/101	94/99/106^∗^	88/91/95^∗^†	92/98/108‡
30 min plasma glucose (mg/dL)	126/143/162	150/168/186^∗^	76/81/88^∗^†	132/163/182‡
1-hour plasma glucose (mg/dL)	106/131/159	146/178/205^∗^	72/92/101^∗^†	112/151/192^∗^‡
2-hour plasma glucose (mg/dL)	96/109/126	105/123/146^∗^	81/92/111^∗^†	90/120/142‡
Fasting insulin (*μ*IU/mL)	3.1/4.3/6.2	2.6/3.3/4.3^∗^	2.8/4.2/4.7	2.4/3.3/6.2
30 min insulin (*μ*IU/mL)	29.2/40.2/58.3	13.7/19.1/24.1^∗^	13.7/20.1/32.9^∗^	0.8/1.4/2.1^∗^†‡
120 min insulin (*μ*IU/mL)	16.3/24.6/39.7	14.5/21.5/34.9^∗^	9.1/15.3/21.9^∗^	10.6/23.0/39.1
HbA1c (%)	5.2 ± 0.3	5.3 ± 0.4^∗^	5.1 ± 0.3†	5.3 ± 0.4
Glucose tolerance				
Normal glucose tolerance (%)	62.2	39.4^∗^	92.9^∗^†	46.7‡
Impaired fasting glucose (%)	32.1	49.8	7.2^∗^†	43.3
Impaired glucose tolerance (%)	13.2	30.9^∗^	2.4	30.0
Current smokers (%)	17.5	25.0^∗^	19.0	20.0
Drinkers (%)	59.9	71.3^∗^	42.9†	63.3
Antihypertensive medications (%)	13.6	14.6	7.1	10.0
Lipid-lowering medications (%)	6.9	7.3	2.4	6.7

Data are expressed as mean ± standard deviation, 25/50/75th percentile values, or %. Characteristics among groups were compared using one-way ANOVA for continuous variables and chi-square tests for categorical variables with Bonferroni correction for multiple comparisons. ^∗^*p* < 0.05 compared to insulinogenic index ≥ 0.4, †*p* < 0.05 compared to insulinogenic index < 0.4, ‡*p* < 0.05 compared to ΔGlu_0–30_ ≤ 0. ΔGlu_0–30_ and ΔIns_0–30_, change in glucose and in insulin, respectively, over the first 30 min on the oral glucose tolerance test.
